# Chelerythrine Chloride Downregulates β-Catenin and Inhibits Stem Cell Properties of Non-Small Cell Lung Carcinoma

**DOI:** 10.3390/molecules25010224

**Published:** 2020-01-06

**Authors:** Win Sen Heng, Shiau-Chuen Cheah

**Affiliations:** 1 Department of Medical Oncology, University of Groningen, University Medical Center Groningen, 9713 GZ Groningen, The Netherlands; 2 Faculty of Medicine and Health Sciences, UCSI University, Kuala Lumpur 56000, Malaysia

**Keywords:** alternative medicine, natural compound, herbal compound, lung cancer, apoptosis, Wnt inhibitor, dosing, benzophenanthridine alkaloid

## Abstract

Plant secondary metabolites have been seen as alternatives to seeking new medicines for treating various diseases. Phytochemical scientists remain hopeful that compounds isolated from natural sources could help alleviate the leading problem in oncology—the lung malignancy that kills an estimated two million people annually. In the present study, we characterized a medicinal compound benzophenanthridine alkaloid, called chelerythrine chloride for its anti-tumorigenic activities. Cell viability assays confirmed its cytotoxicity and anti-proliferative activity in non-small cell lung carcinoma (NSCLC) cell lines. Immunofluorescence staining of β-catenin revealed that there was a reduction of nuclear content as well as overall cellular content of β-catenin after treating NCI-H1703 with chelerythrine chloride. In functional characterizations, we observed favorable inhibitory activities of chelerythrine chloride in cancer stem cell (CSC) properties, which include soft agar colony-forming, migration, invasion, and spheroid forming abilities. Interesting observations in chelerythrine chloride treatment noted that its action abides to certain concentration-specific-targeting behavior in modulating β-catenin expression and apoptotic cell death. The downregulation of β-catenin implicates the downregulation of CSC transcription factors like SOX2 and MYC. In conclusion, chelerythrine chloride has the potential to mitigate cancer growth due to inhibitory actions toward the tumorigenic activity of CSC in lung cancer and it can be flexibly adjusted according to concentration to modulate specific targeting in different cell lines.

## 1. Introduction

In the 21st century, we still very much rely on the testing of phytochemical compounds to facilitate drug discovery. It is not surprising that the next wonder drug hailed by Paul Ehrlich as a “magic bullet” was born through the search from nature. Growing interest sparks on plant-derived compounds because of their medicinally valuable bioactivities [1,2]. Until today, plant products have been used in various aspects of life, most importantly for our daily needs that directly contribute to our well-being. Besides fulfilling our needs in foods and beverages, plant products have historically been used for herbs and remedies for various ailments. Phytochemical compounds are known as plant secondary metabolites. They are plants’ secondary needs that are not involved in their primary metabolism. For this reason, they exist in small concentrations and are unique in molecular structure. This can be useful in two ways in pharmacology: it can be used directly to treat diseases or it can serve as inspiration for novel drug discovery [3]. In addition, natural products have been used for consumption for as long as humans could try and experiment throughout history. This has endowed us with knowledge on which are safe to use and which may be harmful to consume.

Malignancy is not a new problem for mankind. The rate of incidence and death remains high every year, especially for lung cancer—the leading cause of cancer-related death. Statistically, lung cancer comprises 80–85% incidence of NSCLC and 15–20% of small cell lung carcinoma (SCLC); those numbers combined summed to around 2.1 million cases based on 2018′s estimation [4]. Although significant advancements have been observed over the years for NSCLC disease management, some problems remain to be addressed, which include therapeutic resistance, toxicity, suitability, and cost-effectiveness [5,6,7,8,9,10]. From a cellular perspective, CSC’s existence has been proposed to be the underlying factor for therapeutic resistance, relapse, and metastasis due to its role in maintaining and perpetuating the disease [11]. The riddance of CSC may be an essential step toward tumor eradication.

Chelerythrine is a type III benzophenanthridine alkaloid isolated from herbal plants like *Chelidonium majus* and *Macleaya cordata* [12,13,14]. Those herbs have been utilized in folk medicine for various discomforts such as inflammation, pain, and cough, and recently isolated alkaloids including chelerythrine have been demonstrated to possess anti-bacteria, anti-fungal, and anti-tumor activities. At present, chelerythrine is most recognized as a potent inhibitor of protein kinase C (PKC) for isoform α and β [15]. One of the earliest studies to demonstrate chelerythrine’s anti-tumor activity was its cytotoxicity to a wide range of different histologies of cancer cell lines [16]. Studies over the years have successfully established the apoptotic-inducing ability of chelerythrine in a variety of cancer cells, namely in uveal melanoma, leukemia, prostate cancer, hepatoma, and renal cancer [17,18,19,20,21]. The latest reports suggest apoptosis is mediated through reactive oxygen species (ROS)-dependent endoplasmic reticulum (ER) stress induction and possibly through signal transducer and activator of transcription 3 (STAT3) inactivation [22,23]. Studies also showed that chelerythrine was able to inhibit the growth of cisplatin-resistant NSCLC and triple-negative breast cancer cells as well as sensitizing them to chemotherapies [24,25]. All of these suggest that chelerythrine has the potential to target CSC for thorough riddance of tumors.

Wnt/β-catenin is one of the evolutionary-conserved signaling pathways in metazoan that is responsible for regulating cellular processes like proliferation, survival, motility, differentiation, and apoptosis [26]. The pathway is activated upon the binding of Wnt ligand to Frizzled and low-density lipoprotein receptor-related protein 5/6 (LRP5/6) receptors. This association activates a sequential signaling cascade that involves an intracellular transducer like Disheveled to inactivate the constitutively functioning β-catenin degradation complex that constantly targets β-catenin for proteosomal recycling. In the absence of degradation complex activity, free β-catenin molecules translocate to the nucleus to associate with various adaptor proteins and transcription factors, and together they bind onto Wnt responsive elements to transcribe Wnt target genes for regulating the aforementioned cellular processes [27]. During lung development and morphogenesis, the Wnt/β-catenin pathway acts as the speciation control for distal epithelium differentiation [28]. In adult lungs, its role in homeostasis maintenance of the distal epithelium remains consistent as indicated by the responsiveness of AXIN2^+^ alveolar progenitors of the lung toward Wnt signal and the requirement of Wnt signaling in epithelial differentiation of alveolar epithelial type II cells (AEC II) to terminally differentiated AEC I cells [29,30].

In NSCLCs, the Wnt/β-catenin pathway may be aberrantly activated to support tumorigenesis [31,32]. For instance, NSCLCs may self-sustain themselves by overexpressing Wnt ligands. Constitutive expression of β-catenin variants coupled with aberrant cytoplasmic stabilization could increase the activity of β-catenin-mediated transcriptions. In some other case, loss of heterozygosity or hypermethylation of adenomatous polyposis coli (APC) as part of the β-catenin destruction complex, or the presence of its variant, could also contribute to hyperactivation of the Wnt/β-catenin pathway. Alternatively, intracellular Wnt antagonists’ expressions may be reduced or lost [33]. Sustained canonical Wnt activity in NSCLCs leads to maintenance of the CSC component of the tumor, which consequently drives the progression of the disease [34]. Hence, we seek to find out the growth-inhibiting mechanism of chelerythrine in NSCLCs by exploring its inhibitory action on the Wnt/β-catenin pathway. Our findings suggested that chelerythrine chloride indeed possessed some inhibitory activity toward the Wnt/β-catenin pathway through β-catenin downregulation. This inhibition is associated with reduced CSC properties that may potentially lead to improved CSC targeting in the future.

## 2. Results

### 2.1. Cheleythrine Chloride Inhibited the Growth of NCI-H1703, SK-LU-1, and Human Lung Cancer Stem Cells (HLCSC)

**Figure 1:**
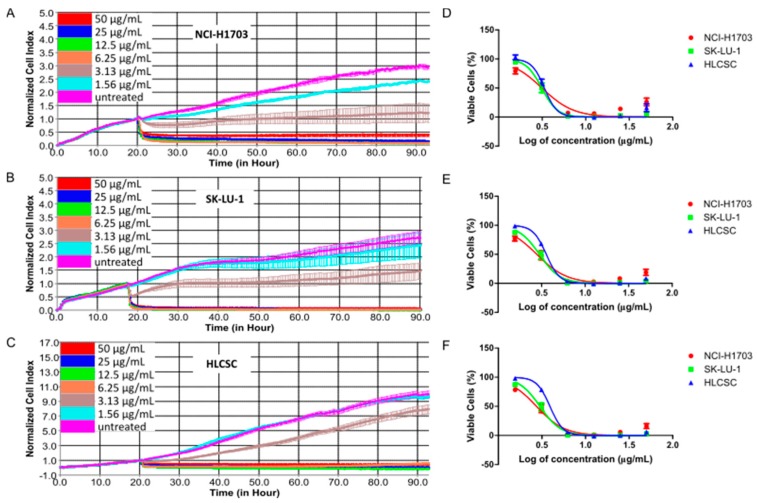
Dose–response curves of chelerythrine chloride treatments in NCI-H1703, SK-LU-1, and human lung cancer stem cells (HLCSC). Representative dose–response curves show the kinetic response of (**A**) NCI-H1703, (**B**) SK-LU-1, and (**C**) HLCSC toward chelerythrine chloride in a span of 72 h treatments. Curves show dose–response of NCI-H1703, SK-LU-1, and HLCSC toward chelerythrine chloride after (**D**) 24, (**E**) 48, and (**F**) 72 h treatment. Dose–response kinetic curves in (**A**–**C**) were plotted from duplicate data obtained in a representative of two independent experiments. Dose–response curves in (**D**–**F**) were derived from means of CI at specified time-points from two independent experiments.

To check whether the growth-inhibitory effect of chelerythrine chloride is applicable to major histological cell types of NSCLC, we used three cell lines consisting of squamous cell carcinoma, adenocarcinoma, and lung CSC cell lines. Using a real-time cell analyzer (RTCA) that evaluates cell viability based on impedance to detect cell attachment, we evaluated chelerythrine chloride’s growth-inhibitory kinetic in NCI-H1703, SK-LU-1, and HLCSC. HLCSC was certified by endorsing companies to exert cancer stem cells properties, including markers expressions (CD133, aldehyde dehydrogenase (ALDH), stage-specific embryonic antigen 3/4 (SSEA3/4), alkaline phosphatase, octamer-binding transcription factor 4 (OCT4) and CD43), and in vivo tumorigenicity (<1000 cells). Figure 1 shows dose–response kinetic curves and dose–response curves for 24, 48, and 72 h treatment of chelerythrine chloride in the three cell lines. The treatment kinetic of chelerythrine chloride always started with rapid initial cytotoxicity indicated by curves with negative gradient, and this cytotoxic effect was more pronouncedly seen in mid-range (6.25 and 12.5 μg/mL) to high-treatment concentrations (25 and 50 μg/mL) in all three cell lines (Figure 1A–C). Growth arresting activity (flat curve) was observed as the after-effect of treatment in mid-range to high concentrations. A closer look at specific time-points after 24 h treatment intervals, NCI-H1703, SK-LU-1, and HLCSC were almost equally sensitive toward chelerythrine chloride at all treatment concentrations (Figure 1D–F). However, minor sensitivity observation from the dose–response curves deduced that NCI-H1703 was slightly more sensitive to 1.56 μg/mL of chelerythrine chloride when compared to SK-LU-1 and HLCSC. This difference can be more clearly seen in differences of IC_20_, IC_50_, and IC_80_ values of chelerythrine chloride among the cell lines (Table 1). Interestingly, NCI-H1703 was slightly more resistant at higher treatment concentrations indicated by slightly higher IC_80_ values. All in all, chelerythrine chloride is a potent growth inhibitor of major histologies of NLCSC.

### 2.2. Chelerythrine Chloride Reduces the Global Expression and the Proportion of Cells with Positive Nuclear β-Catenin

**Figure 2:**
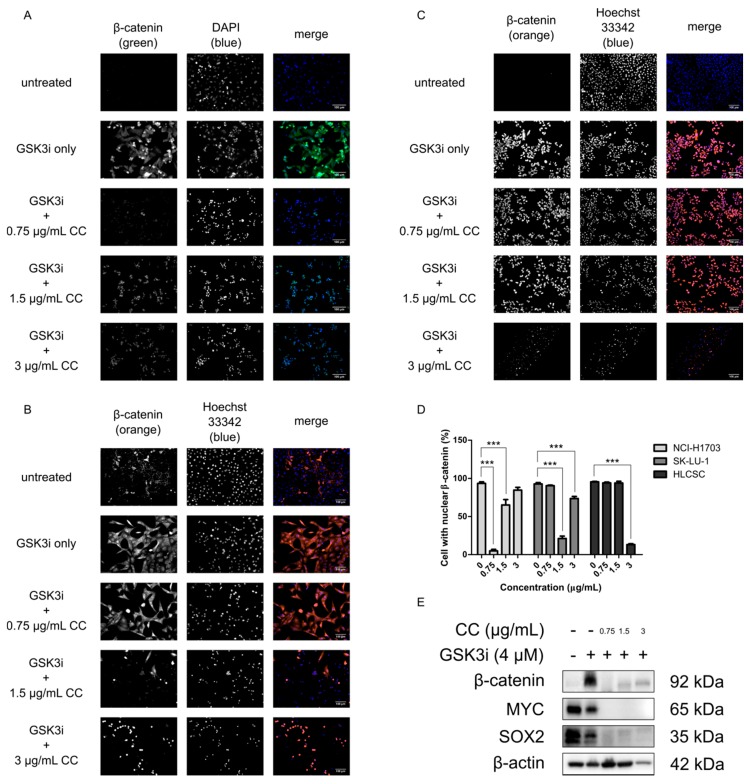
Molecular implications of chelerythrine chloride treatment in lung cancer cell lines. NCI-H1703, SK-LU-1, and HLCSC were treated with GSK3i for 24 h and sequentially treated with various concentrations of chelerythrine chloride for 24 h. Subsequently, immunofluorescence stainings of β-catenin were compared among the cell lines. (**A**) Representative micrographs show immunofluorescence stainings for chelerythrine chloride treatment in NCI-H1703. (**B**) Representative micrographs show immunofluorescence stainings for chelerythrine chloride treatment in SK-LU-1. (**C**) Representative micrographs show immunofluorescence stainings for chelerythrine chloride treatment in HLCSC. Scale bars represent 100 μm. (**D**) Representative quantification shows cell number with positive nuclear β-catenin in an independent immunostaining experiment. Error bars are expressed as mean ± SD. Most responsive chelerythrine chloride-treated cell lines—NCI-H1703′s—protein lysates were resolved using Western blotting. (**E**) Representative Western blots show the effect of chelerythrine chloride treatment toward the expression of CSC-related transcription factors, namely β-catenin, MYC, and SOX2. Statistical significance was expressed as *** *p* < 0.001.

Due to its importance in lung development and homeostasis, as well as its aberrancies during tumorigenesis, the Wnt/β-catenin pathway may be a lucrative target for inhibiting CSCs to ultimately mitigate therapeutic resistance, recurrence, and metastasis [28,30,32,34]. In order to determine whether chelerythrine chloride treatment would impact the signaling of the Wnt/β-catenin pathway, NCI-H1703, SK-LU-1, and HLCSC were treated with chelerythrine chloride and checked for β-catenin expression with immunofluorescence staining. This would not only check the expression level of β-catenin, but also its localization since its nuclear retention is necessary for transduction of Wnt/β-catenin signaling [35,36]. Prior to chelerythrine chloride treatment, GSK3i was added at 4 μM to NCI-H1703 and at 8 μM to SK-LU-1 and HLCSC. GSK3i inhibits GSK3β—the negative regulator of β-catenin degradation machinery to stimulate β-catenin cytoplasmic stabilization and thus enhances nuclear localization. This mimics the phenomenon for negative regulators deregulations and β-catenin nuclear localization and retention during canonical Wnt activation. Visualization of immunofluorescence stainings clearly demonstrated some morphological changes as well as changes in expression level and localization status of β-catenin after chelerythrine chloride treatment (Figure 2A–C). DNA stains (DAPI or Hoechst 33342) revealed that chelerythrine chloride’s treatment may have induced cell shrinkage and chromatin condensation as early signs of apoptotic cell death [37]. These effects were seen at least in two treatment concentrations in all cell lines, namely at 1.5 and 3 μg/mL. Moreover, the stretching pattern of healthy adherent NCI-H1703 and SK-LU-1 cells became more oval-shaped after the treatments. Not only was the average β-catenin cellular expression reduced, but the treatments also diminished the localization of β-catenin in the nucleus. This is indicated by the complete absence between overlapping colors of β-catenin (green or orange) and nuclear site (blue). Statistically, the reduction of cell number with positive nuclear β-catenin was observed in NCI-H1703 at 0.75 and 1.5 μg/mL, in SK-LU-1 at 1.5 and 3 μg/mL, and in HLCSC at 3 μg/mL of chelerythrine chloride treatments (*p* < 0.001, Figure 2D). NCI-H1703 is interestingly more responsive to low concentrations of chelerythrine chloride and due to that we decided to focus on this cell line for downstream experiments. Western blotting on several CSC-related transcription factors including β-catenin revealed that there were downregulations of MYC, SOX2, and β-catenin upon chelerythrine chloride treatments, further suggesting the potential of chelerythrine chloride in impacting CSC activities (Figure 2E). In conclusion, chelerythrine chloride can be specifically concentration-adjusted to inhibit Wnt/β-catenin in different lung cancer cells from different histological origins through β-catenin downregulation. The downregulation may negatively impact CSC properties and induce apoptosis.

### 2.3. Chelerythrine Chloride Partly Induces Apoptosis and Inhibits the Functions of CSC

**Figure 3:**
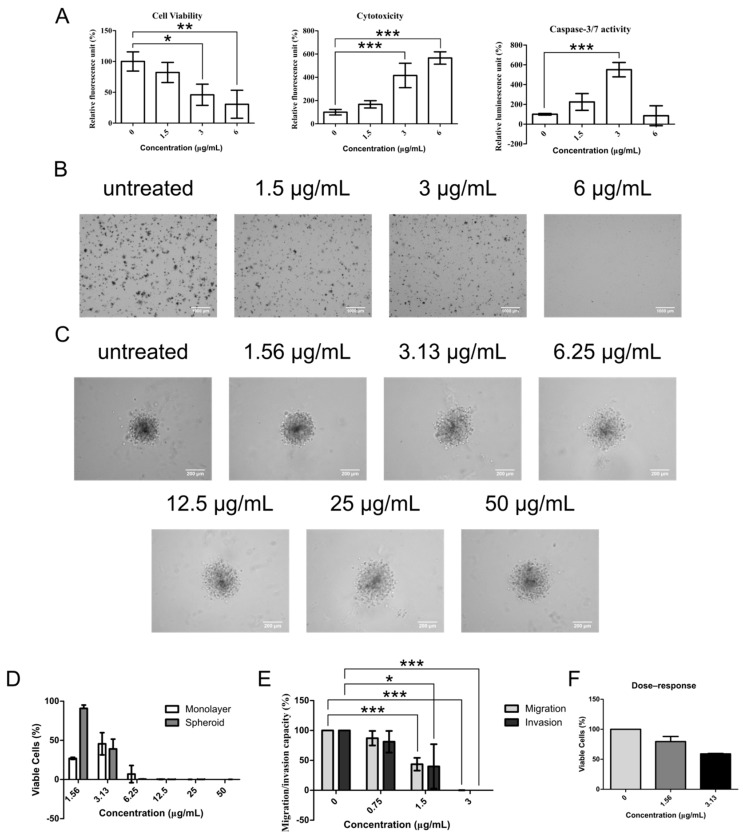
Chelerythrine chloride induces apoptosis and inhibits CSC functions. (**A**) Parallel estimations of cell viability, cytotoxicity and apoptosis were performed after 24 h treatment of chelerythrine chloride in NCI-H1703 at indicated concentrations using ApoTox-Glo triplex assay. (**B**) Representative micrographs show three weeks grown soft agar colonies of NCI-H1703 upon treatment of chelerythrine chloride at indicated concentrations. Scale bars represent 1000 μm. (**C**) Representative micrographs show spheroids’ morphological characteristics after 24 h treatment of chelerythrine chloride at indicated concentrations. Scale bars represent 200 μm. (**D**) Dose–response bar graph show comparison of cytotoxicity of various concentration of chelerythrine chloride between monolayer and spheroid models of NCI-H1703. (**E**) Estimations of migration and invasion of NCI-H1703 after 16 h of chelerythrine chloride treatment at indicated concentrations using real-time cell analyzer (RTCA). (**F**) Effect of corresponding treatment concentrations in (**E**) to cell viability is shown in bar graph. All data were obtained from mean of three independent experiments. Error bars are expressed as mean ± SD. Statistical significance was expressed as *** *p* < 0.001 ** *p* < 0.01; * *p* < 0.05.

The potential of compounds to inhibit CSC activities is best shown by demonstrating its ability to induce apoptosis and its inhibitory action toward CSC functions. ApoTox-Glo triplex assay was first employed to measure cell viability, cytotoxicity, and late apoptotic activity (caspase-3/7 activity) from the same chelerythrine chloride-treated sets of NCI-H1703 cells. Significant reduction of cell viability was observed in 3 and 6 μg/mL (*p* < 0.05 and *p* < 0.01, respectively) of chelerythrine chloride-treated cells (Figure 3A). In parallel with these, cytotoxicity was highly induced in the same treatment groups (*p* < 0.001). Apoptosis signal was detected in both 1.5 and 3 μg/mL treatment groups, but interestingly, only 3 μg/mL-treated cells displayed an elevated level of apoptosis (*p* < 0.001). This suggests that chelerythrine chloride may induce other forms of cell death in the 6 μg/mL treatment group.

The most essential trait of CSCs to perpetuate tumors is by endowing themselves with self-renewal capability. This property can be demonstrated in vitro in soft agar colony-forming assay that determines the efficiency of colony formation under anchorage-independence growth condition. Figure 3B shows visual representations of NCI-H1703 colonies grown in soft agar after being treated with chelerythrine chloride at 1.5, 3, and 6 μg/mL. Generally, chelerythrine chloride-induced dose-dependent inhibitory effect on colony-forming efficiency and complete inhibition was observed in the 6 μg/mL treatment group. Concentration that has very little impact on NCI-H1703′s cell viability, i.e., 1.5 μg/mL, also repressed soft agar formation. Similarly, the dose-dependent inhibitory effect was also observed in cells grown as anchorage-independent multicellular tumor spheroids—a micrometastasis model as shown in Figure 3C. Healthy spheroids grow as compact aggregates, whereas spheroids with compromised health appear loosely packed. Spheroid cell viability assay that is based on ATP production capacity demonstrated a dose-dependent reduction of cell viability in the spheroids upon chelerythrine chloride treatment at increasing concentrations (Figure 3D). Concentration at 1.5 μg/mL again does not appear to interfere with cell growth during the treatment duration of 24 h. However, when estimating cell viability of monolayer NCI-H1703 cells using the same cell viability assay, the 1.5 μg/mL treatment group appears to be more cytotoxic than 3 μg/mL. The discrepancy of this with the estimations from RTCA and ApoTox-Glo cell viability assay may suggest that 1.5 μg/mL of chelerythrine chloride probably interrupts cell ATP production machinery without compromising cell attachment (RTCA) and membrane integrity (ApoTox-Glo cell viability). The effect in spheroid may in general be delayed by a slower diffusion rate of low-concentration chelerythrine chloride.

Besides colony formation, migration and invasion are two of the utmost important traits of surviving CSCs for distant secondary colony formation during therapeutic resistance. Hence it is imperative to determine whether chelerythrine chloride inhibitory effect also affects these properties. Significant decrement of migration and invasion of NCI-H1703 cells was seen after 16 h incubation of chelerythrine chloride at 1.5 μg/mL (migration, *p* < 0.05; invasion, *p* < 0.001) (Figure 3E). Treatment at 3 μg/mL effectively induced complete abolishing of both migration and invasion of NCI-H1703 (migration, *p* < 0.001; invasion, *p* < 0.001). After taking into account the cytotoxic effect of each treatment concentration, chelerythrine chloride retarded approximately 40% of both migration and invasion ability of NCI-H1703 in aforementioned treatment concentrations, respectively (Figure 3E,F).

## 3. Discussion

In this epoch of rapid technological advancement, a portion of the drug development research is still fueled by discoveries from natural resources. From safety perspective, naturally-derived phytochemical compounds are deemed superior, especially when acquired from those which have been incorporated into diet as foods, beverages, spices and even folk medicines for hundreds if not thousands of years [38]. In oncology, some established chemotherapeutic drugs were also derived directly or indirectly from plant secondary metabolites, including paclitaxel, vinorelbine and topotecan [39]. Many phytochemical compounds have been discovered hitherto and characterization works are still ongoing [40].

In the present study, chelerythrine chloride—a benzophenanthridine alkaloid was characterized for its potential to inhibit CSCs and their commonly found properties in NSCLCs. Kinetic profiles of the chelerythrine chloride’s dose–response in three cell lines tested showed an overall similar pattern of cytotoxicity and growth arrest throughout the tested concentrations. We also checked the growth inhibition of chelerythrine chloride in a representative normal cell line—the lung fibroblast IMR-90 cell line, but we did not see selective inhibition of chelerythrine chloride toward cancer cell line (refer to Figures S4 and S5, Spreadsheet S1 and S2 (Supplementary Materials)). However, extrapolations of IC_20_ and IC_80_ values from IC_50_ values revealed interesting behavior of NCI-H1703 toward chelerythrine chloride treatment, i.e., a slight shift to higher sensitivity at low-concentration treatment (<IC_50_ value) and slight shift to higher resistance at high concentration treatment (>IC_50_ value) when compared to the other two cell lines. We also observed similar pattern of higher sensitivity with the significant reduction of β-catenin expression when treated with low, but not high concentrations of chelerythrine chloride—a dose-independent phenomenon that was also seen in the SK-LU-1, but may or may not be the case for HLCSC. Two plausible reasons may be able to explain this specific sensitivity. One reason is that the expression of WNT1 and WNT2 in NCI-H1703 confer dependence toward Wnt/β-catenin signaling for growth [41,42]. However, our immunofluorescence stainings revealed undetectable β-catenin expression in the absence of GSK3i treatment in NCI-H1703, further questioning how Wnt/β-catenin signaling works in NCI-H1703. The other reason explains that NCI-H1703′s squamous cell carcinoma–basal cell cancer identity predisposes sensitivity to SOX2 downregulation as part of the response we saw for chelerythrine chloride treatments [43,44,45]. Co-downregulation, or rather co-upregulation of SOX2 and β-catenin is not a common sight in squamous cell lung carcinoma since they have contradicting regulating behavior in respect to their proximal and distal speciation functions [28,43,46]. Nevertheless, chelerythrine chloride was able to inhibit the growth of lung cancer cell lines from different proximal/distal origins, the β-catenin nuclear localization in several lung histologies, and β-catenin and perhaps SOX2 and MYC expression regardless of the tissue origin. This wide applicability of chelerythrine chloride favors its development as cancer therapy. Furthermore, its sub-to-non-toxic specific targeting potentially offer a new therapeutic window for cancer therapy.

We also saw that only treatment at 3 μg/mL of chelerythrine chloride was able to induce significant increase in apoptotic activity. Albeit insignificant, treatment at 1.5 μg/mL was observed to induce apoptotic cell death as indicated by a two-fold increase of caspase-3/7 activity. Absence of apoptotic activity induction in 6 μg/mL treatment group presumably indicates the occurrence of necrotic cell death as treatment of chelerythrine chloride at 6 μg/mL or higher witnessed a rapid decrease of viable cells. In addition, necrosis is commonly referred as default pathway to cellular death when apoptosis is inhibited, thus making it more likely to be the case [47]. The observation of bimodal cell death occurrence due to chelerythrine treatments is not new as it has been reported by others in leukemia and uveal melanoma [17,18].

Cell death and ROS are two interrelated components. Decades ago, ROS was thought to be insignificant metabolic byproduct that is harmful if not cleared-off or neutralized in timely manner. However, current understandings highlighted the involvement of ROS as an important second messenger for signaling that regulates not only cellular process like cell death, but also proliferation [48]. Modest production of ROS aids in normal functioning of cells, whereas excess amount may lead to lethal oxidative stress. Cellular intrinsic antioxidative machinery normally balances the destructive effect of excessive ROS [49]. In the case of chelerythrine chloride treatment, there could be involvement of ROS generation beyond what the intrinsic cellular antioxidants could handle, thus leading to oxidative stress and cell death. Indeed, several studies including in NSCLC, prostate cancer and renal cell carcinoma confirmed that chelerythrine-induced cell death was mediated through ROS generation and was reversible upon pre-treatment of the ROS scavenging agent—*N*-acetyl-l-cysteine (NAC) [22,23,50]. Different type of cell death can ensue depending on the amount of ROS generated. Apoptosis, autophagic cell death and necrosis are generally induced by low, moderate and high amount of ROS, respectively [48]. In the present study, we observed induction of apoptosis in low concentration of chelerythrine chloride, but not in high, suggesting chelerythrine chloride might dose-dependently generate ROS. This observation was also reported by other investigators [23].

It was hypothesized that during therapeutic resistance, CSCs survive the therapeutic challenge and repopulate tumors locally and perhaps distantly [11]. Equipped with self-renewal ability, they re-establish themselves and the tumor bulk. With their migration and invasion ability, they travel to distant organs as micrometastases to establish a secondary colony. In the present study, chelerythrine chloride was observed to inhibit these multi-steps of relapse and metastasis as shown by the reduction of soft agar colony formation, inhibition of spheroid growth, and reduction of both migration and invasion ability. This suggests that chelerythrine chloride potentially exhibited wide array effects of inhibitory to signaling that regulates cellular processes, in this case including signaling that uses β-catenin, MYC, SOX2, and possibly EMT machinery, which are commonly associated with proliferation, motility, differentiation, and survival.

Chelerythrine chloride treatment modulated great molecular responses in NCI-H1703 even at non-lethal concentrations. Because chelerythrine chloride is a PKC inhibitor, the observed molecular responses may be modulated through PKC inhibition. Indeed, PKC was identified as an additional component of the Wnt/β-catenin pathway that acts to augment GSK3β inhibition by canonical Wnt signaling [51]. Therefore, chelerythrine chloride plausibly abolished GSK3i-induced-β-catenin expression through PKC inhibition. This reasoning is further strengthened by an in vitro observation that confirmed the high affinity of pharmacological targets of chelerythrine chloride (PKC-α and -β) with GSK3β as an inactivating phosphorylation target [52]. The only caveat to this explanation points to the conflicting response of MYC expression as a canonical Wnt downstream activating target [53]. Although GSK3i treatment mimicked the cytoplasmic accumulation and nuclear localization of β-catenin, MYC expression was not upregulated upon treatment. Furthermore, NCI-H1703 already possessed high MYC expression prior to the treatment. Hence, it is not clear whether complete abolishment of MYC is directly influenced by β-catenin downregulation.

As a matter of fact, we are currently still facing a major shortcoming in cancer treatments due to general toxicities. Whether it is due to an excipient or its non-specific cellular targets, even plant-derived chemotherapeutic drugs are still associated with a certain extent of toxicity. Traditional chemotherapy like paclitaxel works by enhancing microtubules’ polymerization and stabilization, hence inducing cytotoxicity through disruption of the normal cell cycle progression during mitosis [54]. The idea of targeting the machinery used during mitosis to induce cytotoxicity is the underlying reason for its non-exclusive targeting to cancer since cell division occurs also in normal cells for homeostasis maintenance [55]. To improve cancer targeting using phytochemical compounds, the targeting may have to be specific, but at the same time flexible. In the present study, we assessed chelerythrine chloride’s effect toward Wnt/β-catenin signaling and we found that chelerythrine chloride can be flexibly adjusted according to concentration to trigger desired specific targeting in different cell lines. Nuclear localization of β-catenin was differently modulated by chelerythrine chloride at different concentrations in different cell lines. This suggests that using the correct concentration, chelerythrine chloride can be used to improve pathway targeting. This specific targeting may not significantly reduce cell viability, but is capable of diminishing self-renewal capacity—a desirable CSC targeting.

A recent study revealed the potential synergistic effect of increasing pathways targeting coverage in combinatorial regimens consisting of phytochemical compounds and chemotherapy [56]. Phytochemical compounds also have the potential to alleviate toxicity induced by chemotherapy, further justifying their usage along with the traditional treatment modalities [55]. Chelerythrine chloride has been thus far combined with erlotinib and cisplatin in pre-clinical studies involving NSCLCs, and respective authors have concluded the desirable synergistic or additive effect of the combined treatment [24,57].

## 4. Materials and Methods

### 4.1. Cell Culture

Human lung squamous cell carcinoma cell line NCI-H1703 and human lung adenocarcinoma cell line SK-LU-1 were purchased from American Type Culture Collections (ATCC, Manassas, VA, USA). Human lung cancer stem cells (HLCSCs) primary cell line was purchased from Celprogen (Torrance, CA, USA). NCI-H1703 and SK-LU-1 were routinely maintained in Gibco’s Dulbecco’s modified Eagle’s medium (DMEM) with 4.5 g/L d-glucose (Thermo Fisher Scientific, Waltham, MA, USA) supplemented with 1% (*v*/*v*) of 100 mM Gibco’s sodium pyruvate, 1% (*v*/*v*) of 10,000 U/mL Penicillin-10,000 μg/mL Streptomycin (Pen-Strep), and 10% (*v*/*v*) of Gibco’s fetal bovine serum (FBS). HLCSC was routinely maintained in human lung cancer stem cell complete growth medium with serum (Celprogen). All cell cultures were maintained in 5% CO_2_ humidified atmospheric condition CO_2_ incubator at 37 °C. Passaging was performed regularly with Sigma-Aldrich’s (St. Louis, MO, USA) 0.25% (*w*/*v*) trypsin-Ethylenediaminetetraacetic acid (EDTA) solution.

### 4.2. Chemicals

Chelerythrine chloride was purchased from Chromadex (Lost Angeles, CA, USA). Dimethyl sulfoxide (DMSO, Sigma-Aldrich, St. Louis, MO, USA) was used to dissolved chelerythrine chloride and GSK-3 inhibitor X (Calbiochem, San Diego, CA, USA) into 10 mg/mL and 1 mg/mL stock solution, respectively.

### 4.3. Cell Viability, Cytotoxicity and Anti-Proliferative Assay

Real-time cell analyzer (RTCA) was performed using an E-plate 16 on xCELLigence RTCA-Dual Purpose (DP) platform (ACEA Bioscience, San Diego, CA, USA). Briefly, 50 μL of complete medium was added into each well for 30 min incubation before acquiring background measurements. Optimized cell density (1 × 10^4^ cells/well for SK-LU-1; 1.25 × 10^4^ cells/well for HLCSC and NCI-H1703) was seeded upon background measurement and was incubated 30 min at room temperature in the biological safety cabinet in order to facilitate cells settling before putting back onto the RTCA-DP platform for overnight incubation. Subsequently, chelerythrine chloride was added to each well and incubated at various concentrations for up to 72 h. RTCA software 2.0 was used to generate kinetic dose–response curve by normalizing the cell index (CI) to the time when the compound was added. IC_50_ values were estimated by plotting CI against concentration using algorithm for sigmoidal dose–response curve with variable slope.

CellTiter-Glo 3D cell viability assay (Promega, Madison, WI, USA) was used to evaluate the growth of spheroid culture (refer to spheroid generation section) along with the parallel run of monolayer culture (seeded at 1 × 10^4^ cells/well). After 24 h treatment with chelerythrine chloride at indicated concentrations, each of the spheroids were transferred to a single well of an opaque-white plate for cell viability measurement according to the manufacturer’s protocol. Monolayer culture was directly seeded in the opaque-white plate. Luminescence was measured as relative luminescence unit (RLU) by using FLUOstar Omega microplate reader (BMG LABTECH, Ortenberg, Germany). Each sample was normalized against respective vehicle control. GraphPad Prism v5.01 software (San Diego, CA, USA) was used to plot concentration against the percentage of viability.

ApoTox-Glo triplex assay (Promega, Madison, WI, USA) was used to confirm cell viability along with cytotoxicity and apoptosis measurement. Apoptosis was measured by monitoring caspase-3/7 activity. Briefly, NCI-H1703 cells were seeded overnight in opaque-walled white microplate at 1 × 10^4^ cells/well before administering chelerythrine chloride at indicated concentrations. After 24 h treatment, the plate was treated according to the manufacturer’s protocol. Sequentially, cell viability and cytotoxicity were evaluated by measuring fluorescence at 400_Ex_/505_Em_ and 485_Ex_/520_Em_, respectively, by using FLUOstar Omega microplate reader. Luminescence for caspase-3/7 activity measurement was then measured upon adding appropriate reagent.

### 4.4. Immunocytochemistry

NCI-H1703, SK-LU-1, and HLCSC were first treated with glycogen synthase kinase 3 inhibitor X (GSK3i) for 24 h upon growing overnight in order to artificially activate the Wnt/β-catenin pathway before the onset of chelerythrine chloride treatment. Immunofluorescence was performed by using Cellomics^®^ Beta-Catenin Activation Kits (Thermo Scientific, Waltham, MA, USA) in accordance with the manufacturer’s protocol or by staining with mouse anti-beta catenin 1 (cat# 610154, BD Biosciences, Franklin Lakes, NJ, USA) and counter-stained with anti-mouse-Alexa Fluor 488 (A-11001, Thermo Fisher Scientific) and DAPI (1:1000 of 2 mg/mL, Sigma-Aldrich). Five random, non-overlapping frames were captured from each well by using Axio Vert.A1 inverted microscope equipped with HXP-120V light source and Axiocam MR R3 camera (Carl Zeiss, Oberkochen, Germany) or EVOS FL cell imaging system (Thermo Fisher Scientific). Cells harboring nuclear β-catenin were manually counted by using Image’s J cell counter plugin and the positive counts were divided by the respective total number of cells in the captured frames to obtain the percentage of cells with positive nuclear β-catenin [58]. Treated samples were compared with untreated Wnt-activated negative control.

### 4.5. SDS-PAGE and Western Blotting

NCI-H1703 was treated with or without GSK3i for 24 h and administered with chelerythrine chloride for the following 24 h. Cells were harvested with rubber policeman and lysed with a mixture of Pierce RIPA buffer (Thermo Fisher Scientific), 1% of 100× Halt Protease Inhibitor (Thermo Fisher Scientific), and 1% of 100× Halt Phosphatase Inhibitor (Thermo Fisher Scientific). Protein quantification was performed using BCA protein assay kit (Thermo Fisher Scientific) following the manufacturer’s protocol and measured using iMark microplate reader (Bio-rad, Basel, Switzerland) at 595 nm. Protein lysate was loaded into SDS-PAGE at 20 μg and transferred onto a PVDF membrane. The membrane was blocked with 5% bovine serum albumin (BSA) prepared in Tris-buffered saline (TBS) containing 0.05% Tween-20 (TBST). Primary antibodies mouse anti-beta catenin 1 (cat# 610154, BD Biosciences), rabbit anti-MYC (cat# 5605, Cell Signaling Technology Inc., Danvers, MA, USA), and mouse anti-SOX2 (cat# 4900, Cell Signaling Technology Inc.) were applied overnight at 4 °C at 1:1000. Upon incubation, TBST was used to wash unbound antibodies and subsequently applied with polyclonal HRP-conjugated goat anti-rabbit (cat# P0448, Dako, Jena, Germany) or rabbit anti-mouse (P0260, Dako) secondary antibodies at 1:2000 for 1 h at room temperature. Chemiluminescent signal was resolved using lumi-light plus Western blotting substrate (Roche, Basel, Switzerland) and visualized using ChemiDoc MP imaging system (Bio-rad). Mouse anti-actin (cat# 8691002, MP Biomedicals, Santa Ana, CA, USA) was used as loading control at 1:10,000.

### 4.6. Soft Agar Colony Formation Assay

The assay was performed as described previously with some modifications [59]. Briefly, top/bottom agar was established at 0.35%/0.6% (*w*/*v*) with noble agar (BD Biosciences, Franklin Lakes, NJ, USA) in a 6-well plate. NCI-H1703 cells were suspended within the top agar at 5 × 10^4^ cells. Treated and untreated groups were kept wet with either medium or medium containing chelerythrine chloride at adjusted concentrations by a renewal of every three days. After three weeks, the plate was collected and stained with 0.01% (*v*/*v*) of crystal violet for 30 min at room temperature. The plate was then washed three times with PBS. SZ51 zoom stereo microscope (Olympus, Shinjuku, Tokyo, Japan) was used to visualize the colonies, and toupcam microscope digital camera (ToupTek, Zhejiang, China) equipped with Toupview software (ToupTek, Zhejiang, China) was used to capture the micrographs.

### 4.7. Spheroid Generation

NCI-H1703 was seeded at 400 cells/well in a Costar ultra-low attachment microplate (Corning, New York, NY, USA) by using the same DMEM medium used for monolayer culture. After an overnight incubation, matrigel (final concentration at 2.5% (*v*/*v*)) was added gently toward the center of the well to facilitate the compact formation of a spherical structure. The spheroid was optimized to grow to 300–400 μm in diameter four days post-generation. The diameter of the spheroid was measured by using Zen 2 pro software (Carl Zeiss, Oberkochen, Germany).

### 4.8. Migration and Invasion Assay

RTCA-DP system was similarly used for migration and invasion evaluation. CIM-plate 16 that consists of two chambers was used for this purpose. Overnight serum-starved 4 × 10^4^ NCI-H1703 cells were seeded with serum-free medium at the upper chamber upon background measurement, whereas medium containing 10% (*v*/*v*) of FBS was added to the lower chamber to serve as chemoattractant. Serum-free medium in the lower chamber served as control. For migration, no matrigel coating is necessary, but for invasion, 1:30 matrigel was used to coat the upper chamber 4 h prior to cell seeding. Chelerythrine chloride was added at the indicated concentration upon seeding. The migration–invasion was monitored for up to 24 h. RTCA software 2.0 was used to generate dose–response kinetic curve of both migration and invasion of NCI-H1703 by expressing CI against time.

### 4.9. Statistical Analysis

All data were expressed as mean ± standard deviation (SD). Statistical significance was evaluated by using one-way analysis of variance (ANOVA) in GraphPad Prism v5.01 software. Dunnet’s multiple comparison test was used for post-hoc. Statistical significance was expressed as *** *p* < 0.001 ** *p* < 0.01 * *p* < 0.05. All data were collected from three independent experiments unless otherwise specified.

## 5. Conclusions

With our characterizations, we have confirmed that chelerythrine chloride exhibits cytotoxicity and an anti-proliferative effect in lung cancer cell lines. A specific effect like rapid cell death, which implies necrotic default cellular death, is typically induced at high concentrations. At lower concentrations, apoptosis might ensue. Concentration modulation also shifts sensitivity toward certain pharmacological actions such as the reduction of nuclear β-catenin localization observed in our study. Such concentration modulation is cell line-dependent. The current view for the usage of phytochemical compounds is in combination with chemotherapy. The usage of phytochemical compounds in cancer therapy will need to focus on manipulating their minimum effective dose and maximum tolerable dose. In pharmacology, dosing may be the key in improving the pharmacokinetics issue, but the current study also highlights that it can also enhance pharmacodynamics, particularly on how specific targeting is desired. With this specific targeting possibility, dosing in pharmacokinetics and pharmacodynamics becomes a compounded challenge. Further study thus needs to embark on how dosing can be manipulated through these two pharmacological aspects.

## Figures and Tables

**Table 1:** IC_20_, IC_50_, and IC_80_ values of chelerythrine chloride.

**Cell Lines**	**IC_20_ (μg/mL) ^1^**		
	**24 h**	**48 h**	**72 h**
NCI-H1703	1.85 ± 0.04	1.81 ± 0.03	1.89 ± 0.05
SK-LU-1	2.35 ± 0.02	2.17 ± 0.04	2.18 ± 0.04
HLCSC	2.54 ± 0.08	2.73 ± 0.05	3.04 ± 0.08
**Cell Lines**	**IC_50_ (μg/mL) ^2^**		
	**24 h**	**48 h**	**72 h**
NCI-H1703	3.30 ± 0.08	3.16 ± 0.05	3.15 ± 0.08
SK-LU-1	3.14 ± 0.03	3.17 ± 0.06	3.19 ± 0.06
HLCSC	3.20 ± 0.10	3.48 ± 0.06	3.88 ± 0.10
**Cell Lines**	**IC_80_ (μg/mL) ^1^**		
	**24 h**	**48 h**	**72 h**
NCI-H1703	5.87 ± 0.14	5.48 ± 0.09	5.23 ± 0.13
SK-LU-1	4.19 ± 0.04	4.62 ± 0.08	4.65 ± 0.09
HLCSC	4.04 ± 0.12	4.43 ± 0.08	4.94 ± 0.13

^1^ IC_20/80_ values were estimated using the formula ${\mathrm{IC}}_{F}=(\frac{100-F}{F}{)}^{1/H}\;\times \;{\mathrm{IC}}_{50}$, whereby F is the desired percentage of maximal inhibition, i.e., 20% or 80%, and H is hill slope/gradient of the non-linear regression curve. ^2^ IC_50_ data were based on mean values obtained from duplicated dose–response curves in two independent experiments.

## Supplementary Materials

The following are available online: Figure S1: kinetic dose–response curve of NCI-H1703 in independent experiment 2; Figure S2: kinetic dose–response curve of SK-LU-1 in independent experiment 2; Figure S3: kinetic dose–response curve of HLCSC in independent experiment 2; Figure S4: kinetic dose–response curve of IMR-90 in independent experiment 1; Figure S5: kinetic dose–response curve of IMR-90 in independent experiment 2; Figure S6: compilation of soft agar micrographs after CC treatment; Figure S7: compilation of western blots of MYC, β-catenin, and SOX2 in NCI-H1703 after CC treatment; Spreadsheet S1: raw data for dose–response curve of chelerythrine chloride; Spreadsheet S2: raw data for IC_20_, IC_50_, and IC_80_ values of chelerythrine chloride; Spreadsheet S3: raw data for quantification of cell count with positive nuclear β-catenin after CC treatment; Spreadsheet S4: cell viability, cytotoxicity, and apoptosis after CC treatment as evaluated using ApoTox-Glo; Spreadsheet S5: dose–response of CC in spheroid vs. monolayer model of NCI-H1703; Spreadsheet S6: dose–response, migration, and invasion of NCI-H1703 after CC treatment.

## References

[1] Altemimi A., Lakhssassi N., Baharlouei A., Watson D.G., Lightfoot D.A. (2017). Phytochemicals: Extraction, isolation, and identification of bioactive compounds from plant extracts. Plants.

[2] Son N.T. (2019). Genus *Miliusa*: A review of phytochemistry and pharmacology. Evid.-Based Complement. Altern. Med..

[3] Guo Z. (2017). The modification of natural products for medical use. Acta Pharm Sin. B.

[4] Bray F., Ferlay J., Soerjomataram I., Siegel R.L., Torre L.A., Jemal A. (2018). Global cancer statistics 2018: GLOBOCAN estimates of incidence and mortality worldwide for 36 cancers in 185 countries. CA Cancer J. Clin..

[5] Ruiz-Ceja K.A., Chirino Y.I. (2017). Current FDA-approved treatments for non-small cell lung cancer and potential biomarkers for its detection. Biomed. Pharm..

[6] Kim E.S. (2016). Chemotherapy resistance in lung cancer. Adv. Exp. Med. Biol..

[7] Sosa Iglesias V., Giuranno L., Dubois L.J., Theys J., Vooijs M. (2018). Drug resistance in non-small cell lung cancer: A potential for NOTCH targeting?. Front. Oncol..

[8] Remesh A. (2017). Toxicities of anticancer drugs and its management. Int. J. Basic Clin. Pharm..

[9] Johnson D.B., Sullivan R.J., Menzies A.M. (2017). Immune checkpoint inhibitors in challenging populations. Cancer.

[10] Seung S.J., Hurry M., Hassan S., Walton R.N., Evans W.K. (2019). Cost-of-illness study for non-small-cell lung cancer using real-world data. Curr. Oncol..

[11] Heng W.S., Gosens R., Kruyt F.A.E. (2019). Lung cancer stem cells: Origin, features, maintenance mechanisms and therapeutic targeting. Biochem. Pharm..

[12] Han N., Yang Z., Liu Z., Liu H., Yin J. (2016). Research progress on natural benzophenanthridine alkaloids and their pharmacological functions: A review. Nat. Prod. Commun..

[13] Ni H., Martínez Y., Guan G., Rodríguez R., Más D., Peng H., Valdivié Navarro M., Liu G. (2016). Analysis of the impact of isoquinoline alkaloids, derived from *Macleaya cordata* extract, on the development and innate immune response in swine and poultry. BioMed Res. Int..

[14] Wei Q., Zhao M., Li X. (2017). Extraction of chelerythrine and its effects on pathogenic fungus spore germination. Pharm. Mag..

[15] Herbert J.M., Augereau J.M., Gleye J., Maffrand J.P. (1990). Chelerythrine is a potent and specific inhibitor of protein kinase C. Biochem. Biophys. Res. Commun..

[16] Chmura S.J., Dolan M.E., Cha A., Mauceri H.J., Kufe D.W., Weichselbaum R.R. (2000). In vitro and in vivo activity of protein kinase c inhibitor chelerythrine chloride induces tumor cell toxicity and growth delay in vivo. Clin. Cancer Res..

[17] Kemény-Beke Á., Aradi J., Damjanovich J., Beck Z., Facskó A., Berta A., Bodnár A. (2006). Apoptotic response of uveal melanoma cells upon treatment with chelidonine, sanguinarine and chelerythrine. Cancer Lett..

[18] Vrba J., Doležel P., Vičar J., Modrianský M., Ulrichová J. (2008). Chelerythrine and dihydrochelerythrine induce G1 phase arrest and bimodal cell death in human leukemia HL-60 cells. Toxicol. Vitr..

[19] Malíková J., Zdařilová A., Hlobilková A., Ulrichová J. (2006). The effect of chelerythrine on cell growth, apoptosis, and cell cycle in human normal and cancer cells in comparison with sanguinarine. Cell Biol. Toxicol..

[20] Zhang Z., Guo Y., Zhang J., Wei X. (2011). Induction of apoptosis by chelerythrine chloride through mitochondrial pathway and Bcl-2 family proteins in human hepatoma SMMC-7721 Cell. Arch. Pharm. Res..

[21] Chen X.-M., Zhang M., Fan P.-L., Qin Y.-H., Zhao H.-W. (2016). Chelerythrine chloride induces apoptosis in renal cancer HEK-293 and SW-839 cell lines. Oncol. Lett..

[22] Wu S., Yang Y., Li F., Huang L., Han Z., Wang G., Yu H., Li H. (2018). Chelerythrine induced cell death through ROS-dependent ER stress in human prostate cancer cells. Onco Targets.

[23] He H., Zhuo R., Dai J., Wang X., Huang X., Wang H., Xu D. (2019). Chelerythrine induces apoptosis via ROS-mediated endoplasmic reticulum stress and STAT3 pathways in human renal cell carcinoma. J. Cell. Mol. Med..

[24] Gao Z., Han B., Sha H., Shi Z., Yang X., Feng J. (2010). Effects of a protein kinase C inhibitor combined with cisplatin on non-small cell lung cancer. Zhonghua Jie He He Hu Xi Za Zhi.

[25] Lin W., Huang J., Yuan Z., Feng S., Xie Y., Ma W. (2017). Protein kinase C inhibitor chelerythrine selectively inhibits proliferation of triple-negative breast cancer cells. Sci. Rep..

[26] Willert K., Jones K.A. (2006). Wnt signaling: Is the party in the nucleus?. Genes Dev..

[27] Kim W., Kim M., Jho E. (2013). Wnt/β-catenin signalling: From plasma membrane to nucleus. Biochem. J..

[28] Mucenski M.L., Wert S.E., Nation J.M., Loudy D.E., Huelsken J., Birchmeier W., Morrisey E.E., Whitsett J.A. (2003). β-Catenin is required for specification of proximal/distal cell fate during lung morphogenesis. J. Biol. Chem..

[29] Frank D.B., Peng T., Zepp J., Snitow M., Vincent T., Penkala I.J., Cui Z., Herriges M.J., Morley M.P., Zhou S. (2016). Emergence of a wave of Wnt signaling that regulates lung alveologenesis through controlling epithelial self-renewal and differentiation. Cell Rep..

[30] Zacharias W.J., Frank D.B., Zepp J.A., Morley M.P., Alkhaleel F.A., Kong J., Zhou S., Cantu E., Morrisey E.E. (2018). Regeneration of the lung alveolus by an evolutionarily conserved epithelial progenitor. Nature.

[31] Stewart D.J. (2014). Wnt signaling pathway in non–small cell lung cancer. JNCI J. Natl. Cancer Inst..

[32] Rapp J., Jaromi L., Kvell K., Miskei G., Pongracz J.E. (2017). WNT signaling—Lung cancer is no exception. Respir. Res..

[33] Song Z., Wang H., Zhang S. (2019). Negative regulators of Wnt signaling in non-small cell lung cancer: Theoretical basis and therapeutic potency. Biomed. Pharm..

[34] Tammela T., Sanchez-Rivera F.J., Cetinbas N.M., Wu K., Joshi N.S., Helenius K., Park Y., Azimi R., Kerper N.R., Wesselhoeft R.A. (2017). A Wnt-producing niche drives proliferative potential and progression in lung adenocarcinoma. Nature.

[35] Krieghoff E., Behrens J., Mayr B. (2006). Nucleo-cytoplasmic distribution of β-catenin is regulated by retention. J. Cell Sci..

[36] Lu Y., Xie S., Zhang W., Zhang C., Gao C., Sun Q., Cai Y., Xu Z., Xiao M., Xu Y. (2017). Twa1/Gid8 is a β-catenin nuclear retention factor in Wnt signaling and colorectal tumorigenesis. Cell Res..

[37] Elmore S. (2007). Apoptosis: A review of programmed cell death. Toxicol. Pathol..

[38] Veeresham C. (2012). Natural products derived from plants as a source of drugs. J. Adv. Pharm. Technol. Res..

[39] Iqbal J., Abbasi B.A., Mahmood T., Kanwal S., Ali B., Shah S.A., Khalil A.T. (2017). Plant-derived anticancer agents: A green anticancer approach. Asian Pac. J. Trop. Biomed..

[40] Mathur S., Hoskins C. (2017). Drug development: Lessons from nature. Biomed. Rep..

[41] He B., You L., Uematsu K., Xu Z., Lee A.Y., Matsangou M., McCormick F., Jablons D.M. (2004). A monoclonal antibody against WNT-1 induces apoptosis in human cancer cells. Neoplasia.

[42] You L., He B., Xu Z., Uematsu K., Mazieres J., Mikami I., Reguart N., Moody T.W., Kitajewski J., McCormick F. (2004). Inhibition of Wnt-2-mediated signaling induces programmed cell death in non-small-cell lung cancer cells. Oncogene.

[43] Ochieng J.K., Schilders K., Kool H., Boerema-De Munck A., Buscop-Van Kempen M., Gontan C., Smits R., Grosveld F.G., Wijnen R.M.H., Tibboel D. (2014). Sox2 regulates the emergence of lung basal cells by directly activating the transcription of Trp63. Am. J. Respir. Cell Mol. Biol..

[44] Kim B.R., de Laar E.V., Cabanero M., Tarumi S., Hasenoeder S., Wang D., Virtanen C., Suzuki T., Bandarchi B., Sakashita S. (2016). SOX2 and PI3K cooperate to induce and stabilize a squamous-committed stem cell injury state during lung squamous cell carcinoma pathogenesis. PLoS Biol..

[45] Ferone G., Song J.-Y., Sutherland K.D., Bhaskaran R., Monkhorst K., Lambooij J.-P., Proost N., Gargiulo G., Berns A. (2016). SOX2 is the determining oncogenic switch in promoting lung squamous cell carcinoma from different cells of origin. Cancer Cell.

[46] Hashimoto S., Chen H., Que J., Brockway B.L., Drake J.A., Snyder J.C., Randell S.H., Stripp B.R. (2012). β-Catenin–SOX2 signaling regulates the fate of developing airway epithelium. J. Cell Sci..

[47] Golstein P., Kroemer G. (2007). Cell death by necrosis: Towards a molecular definition. Trends Biochem. Sci..

[48] Covarrubias L., Hernández-García D., Schnabel D., Salas-Vidal E., Castro-Obregón S. (2008). Function of reactive oxygen species during animal development: Passive or active?. Dev. Biol..

[49] Milkovic L., Cipak Gasparovic A., Cindric M., Mouthuy P.-A., Zarkovic N. (2019). Short overview of ROS as cell function regulators and their implications in therapy concepts. Cells.

[50] Tang Z.-H., Cao W.-X., Wang Z.-Y., Lu J.-H., Liu B., Chen X., Lu J.-J. (2017). Induction of reactive oxygen species-stimulated distinctive autophagy by chelerythrine in non-small cell lung cancer cells. Redox Biol..

[51] Chen R.-H., Ding W.V., McCormick F. (2000). Wnt Signaling to β-catenin Involves Two Interactive Components glycogen synthase kinase-3β inhibition and activation of protein kinase c. J. Biol. Chem..

[52] Goode N., Hughes K., Woodgett J.R., Parker P.J. (1992). Differential regulation of glycogen synthase kinase-3 beta by protein kinase C isotypes. J. Biol. Chem..

[53] He T.C., Sparks A.B., Rago C., Hermeking H., Zawel L., da Costa L.T., Morin P.J., Vogelstein B., Kinzler K.W. (1998). Identification of c-MYC as a target of the APC pathway. Science.

[54] Weaver B.A. (2014). How taxol/paclitaxel kills cancer cells. Mol. Biol. Cell.

[55] Sak K. (2012). Chemotherapy and dietary phytochemical agents. Chemother. Res. Pr..

[56] Chamberlin S.R., Blucher A., Wu G., Shinto L., Choonoo G., Kulesz-Martin M., McWeeney S. (2019). Natural product target network reveals potential for cancer combination therapies. Front. Pharm..

[57] He M., Yang Z., Zhang L., Song C., Li Y., Zhang X. (2017). Additive effects of cherlerythrine chloride combination with erlotinib in human non-small cell lung cancer cells. PLoS ONE.

[58] Schneider C.A., Rasband W.S., Eliceiri K.W. (2012). NIH Image to ImageJ: 25 years of image analysis. Nat. Meth..

[59] Baranwal S., Wang Y., Rathinam R., Lee J., Jin L., McGoey R., Pylayeva Y., Giancotti F., Blobe G.C., Alahari S.K. (2011). Molecular Characterization of the Tumor-Suppressive Function of Nischarin in Breast Cancer. JNCI J. Natl. Cancer Inst..

