# Supplementary figures and images for: Chelerythrine Chloride Downregulates β-Catenin and Inhibits Stem Cell Properties of Non-Small Cell Lung Carcinoma
Source: Molecules. 2020 Jan 6;25(1):224. doi: 10.3390/molecules25010224 (PMC6983151; doi:10.3390/molecules25010224)

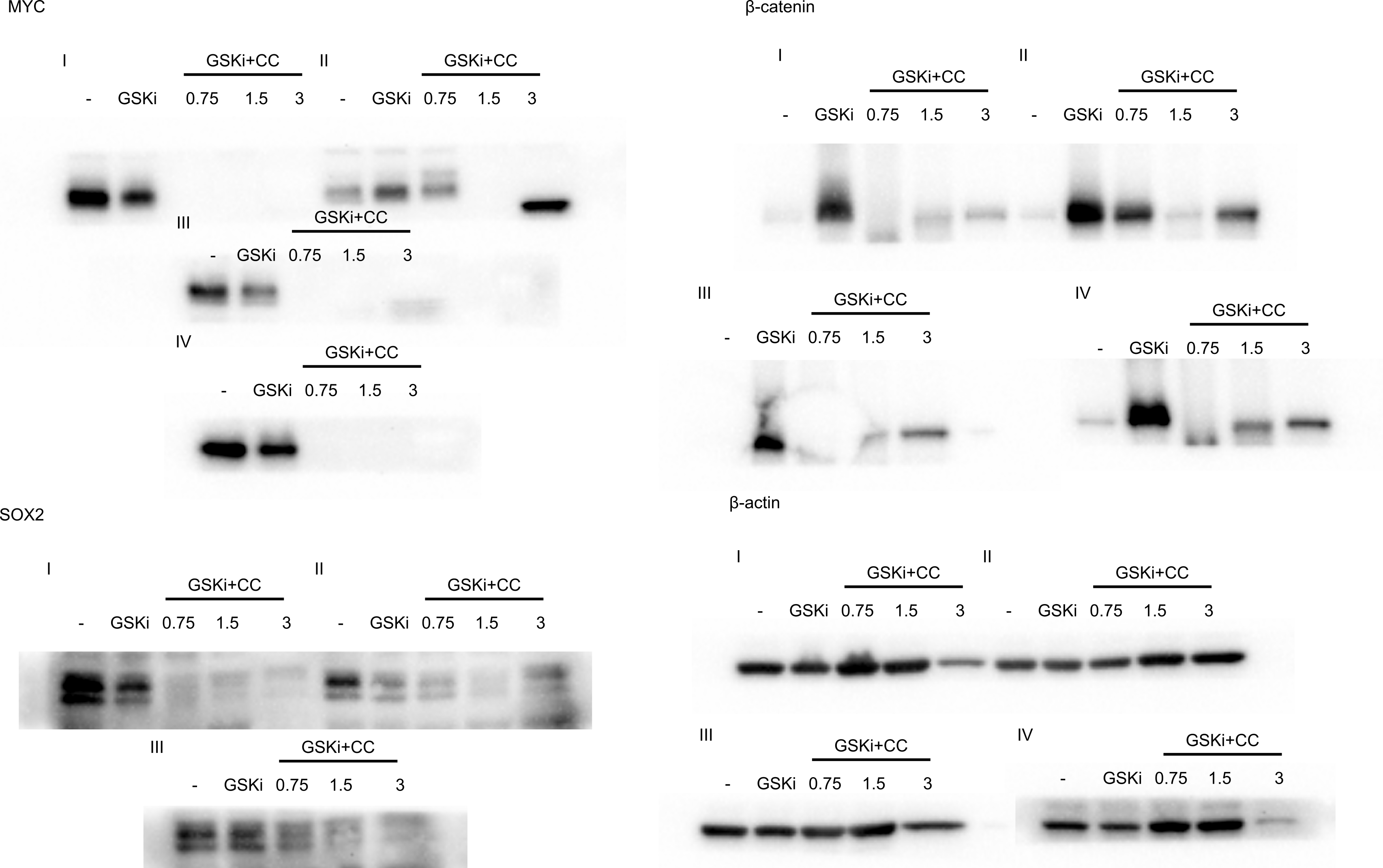

Supplement: Supplementary file 1 [file molecules-25-00224-s001.zip › Figure S7-compilation of western blots of MYC, β-catenin and SOX2 in NCI-H1703 after CC treatment.png]
